# Electromagnetic Properties of Carbon Nanotube/BaFe_12−x_Ga_x_O_19_/Epoxy Composites with Random and Oriented Filler Distributions

**DOI:** 10.3390/nano11112873

**Published:** 2021-10-28

**Authors:** Olena S. Yakovenko, Lyudmila Yu. Matzui, Ludmila L. Vovchenko, Victor V. Oliynyk, Volodymyr V. Zagorodnii, Sergei V. Trukhanov, Alex V. Trukhanov

**Affiliations:** 1 Physics Department, Taras Shevchenko National University of Kyiv, Volodymyrska Str. 64/13, 01601 Kyiv, Ukraine; svtruhanov@yandex.ru (O.S.Y.); l_matzui@mail.ru (L.Y.M.); ll_vovchenko@mail.ru (L.L.V.); vv_oliynyk@mail.ru (V.V.O.); vv_zagorodnii@mail.ru (V.V.Z.); 2 Department of Technology of Electronics Materials, National University of Science and Technology “MISiS”, Leninskii av., 4119049 Moscow, Russia; sv_truhanov@mail.ru; 3 Laboratory of Magnetic Films Physics, Scientific-Practical Materials Research Centre of National Academy of Sciences of Belarus, 220072 Minsk, Belarus

**Keywords:** doped M-type hexaferrites, carbon-based magnetodielectric nanocomposites, microwave properties, natural ferromagnetic resonance, resonance frequency

## Abstract

The microwave properties of epoxy composites filled with 30 wt.% of BaFe_12–*x*_Ga*_x_*O_19_ (0.1 ≤ *x* ≤ 1.2) and with 1 wt.% of multi-walled carbon nanotubes (CNTs) were investigated in the frequency range 36–55 GHz. A sufficient increase in the microwave shielding efficiency was found for ternary 1 wt.%CNT/30 wt.% BaFe_12–*x*_Ga*_x_*O_19_/epoxy composites compared with binary 1% CNT/epoxy and 30 wt.% BaFe_12–*x*_Ga*_x_*O_19_/epoxy due to the complementary contributions of dielectric and magnetic losses. Thus, the addition of only 1 wt.% of CNTs along with 30 wt.% of barium hexaferrite into epoxy resin increased the frequency range where electromagnetic radiation is intensely attenuated. A correlation between the cation Ga^3+^ concentration in the BaFe_12–*x*_Ga*_x_*O_19_ filler and amplitude–frequency characteristics of the natural ferromagnetic resonance (NFMR) in 1 wt.%CNT/30 wt.% BaFe_12–*x*_Ga*_x_*O_19_/epoxy composites was determined. Higher values of the resonance frequency $f_{res}$ (51.8–52.4 GHz) and weaker dependence of $f_{res}$ on the Ga^3+^ concentration were observed compared with pressed polycrystalline BaFe_12–*x*_Ga*_x_*O_19_ ($f_{res}$ = 49.6–50.4 GHz). An increase in the NFMR amplitude on the applied magnetic field for both random and aligned 1 wt.% CNT/30 wt.% BaFe_12–*x*_Ga*_x_*O_19_/epoxy composites was found. The frequency of NFMR was approximately constant in the range of the applied magnetic field, H = 0–5 kOe, for the random 1 wt.% CNT/30 wt.% BaFe_12–*x*_Ga*_x_*O_19_/epoxy composite, and it slightly increased for the aligned 1 wt.% CNT/30 wt.% BaFe_12–*x*_Ga*_x_*O_19_/epoxy composite.

## 1. Introduction

Composites are considered multifunctional materials having suitable structural, microstructural, magnetic, electromagnetic, and other properties for certain applications. In particular, composites work as a material for protective coatings and shields which could be applied as microwave absorbers. Investigation of microwave absorbing materials is important since such developments allow product appliances that reduce electromagnetic interference, protecting devices and biological tissues from undesirable radiation. Electromagnetic energy can be absorbed completely when magnetic and dielectric losses are combined in the material. Microwave absorbers are effective when electromagnetic impedance matching and attenuation of electromagnetic waves are achieved within the material. Improving the effectiveness of microwave absorbing materials is possible by changing their magnetic, conductive, or dielectric components. The current trend is the manufacture of composites with a hybrid filler, which allows the benefits of different components to be combined [1,2,3]. Therefore, many studies are devoted to the research of the microwave absorption properties of composites with different types of fillers. Among them are nickel-coated carbon fibers and MWCNTs [4], carbonyl-iron powder and carbon black [5], graphite nanoplatelets and carbonyl iron [6], etc. Many papers are devoted to composites with hexaferrites and their derivatives, as well as to composites with carbon materials. Adding graphene derivatives to a magnetic/polymer composite can increase both the reflection loss and the absorbing bandwidth arising from the synergy of dielectric loss and magnetic loss. Ferrites, which have high coercive force and saturation magnetization, act as traditional nano-absorbing materials [7,8,9]. In [10], the microwave absorption properties of composites with carbon fiber/Fe_3_O_4_ and graphene/BaFe_12_O_19_/Fe_3_O_4_ were studied, and the analysis showed that the presence of non-magnetic carbon fiber and graphene causes a significant reduction in coercivity while maintaining reasonable saturation and remnant magnetization, thereby improving the microwave absorption capability of the prepared composites. Therefore, combining fillers such as carbon nanotubes (dielectric component) and hexaferrites (magnetic component) serves to improve the electromagnetic response of composite materials [11,12,13]. With the exception of a high absorption intensity and a wide absorption bandwidth, such composites could be thin and lightweight [14]. Additionally, advantages such as low cost, easy preparation, large magnetocrystalline anisotropy, high coercivity, high Curie temperature, and high magnetic loss are characteristic of hexaferrites [15,16,17,18], meaning the incidence of electromagnetic radiation can be reduced as much as possible in hexaferrite-based composites. The natural ferrimagnetic resonance frequency of M-type hexagonal ferrite BaFe_12_O_19_ is about 50 GHz [7], while for ferrites with substituted ions, the shift in the resonance frequency depends on the substitution level. This fact opens up perspectives of tailored optimization of the composite nano-structure for microwave applications. In [19], the effect of Ti substitution on the static and microwave magnetic properties of composites with BaFe_12−x_Ti_x_O_19_ was discussed. Multi-nanolayer structures showed high perspectives relative to microwave absorption performance. Thus, single-layer and multilayer samples were investigated in detail in [20], where absorbers with BaFe_12_O_19_ and BaCoZnFe_10_O_19_ layers of different thicknesses were designed. The absorber was optimized due to the combination of nanolayers; a reflection loss of less than −30 dB (99.9% absorption) for layer thicknesses of less than 500 nm was achieved [19].

Many articles discuss the achievement of higher performance in terms of the absorption properties of composites in the X and K_u_ bands [21]. Li, Jun et al. investigated co-substituted hexaferrites and their microwave absorption capacity at lower frequencies [22]. Incorporation of a spiraled MWCNTs/BaFe_12_O_19_ hybrid into epoxy resin showed the highest microwave absorption of more than 99.9%, with a minimum reflection loss of − 43.99 dB and an absorption bandwidth of 2.56 GHz [23]. The values of the real and imaginary parts of the permittivity of BaCu_x_Mg_x_Zr_2x_Fe_12−4x_O_19_/MWCNTs nanocomposites with different substitutions were much higher than those of the corresponding samples without MWCNTs [12].

In addition, it should be noted that due to the rapid development of technology, the frequency range in which such composites operate must be expanded to higher frequencies. However, such works which present the results of studies of the microwave properties of composites at frequencies above 18 GHz are few. Not only the composition but also the method of manufacturing the composite allows varying the properties of the obtained material. Thus, composites with an ordered distribution of fillers exhibit improved properties, including higher electrical conductivity and dielectric permittivity [24].

The aim of this work was to study the effect of the addition of carbon nanotubes on the electromagnetic properties of epoxy composites filled with substituted hexaferrites BaFe_12–x_Ga_x_O_19_ (0.1 < x < 1.2) in the frequency range 36–55 GHz.

## 2. Materials and Methods

**Figure 1:**
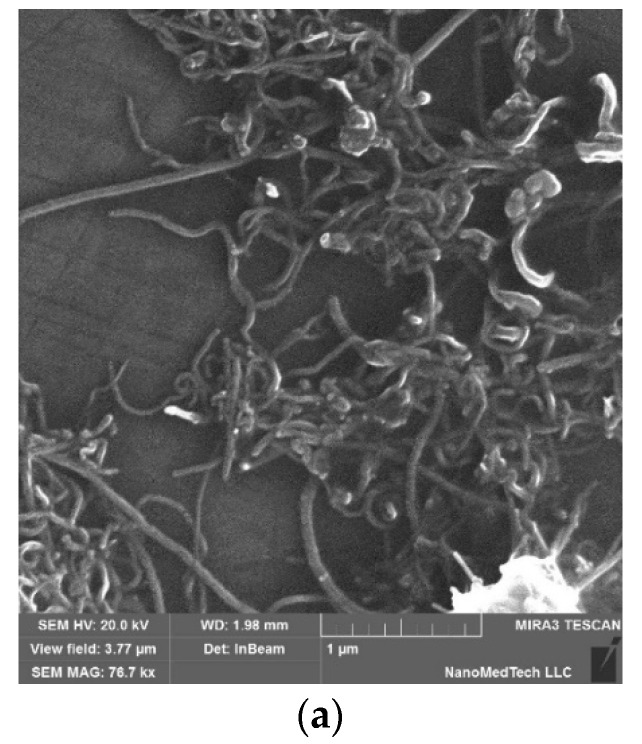

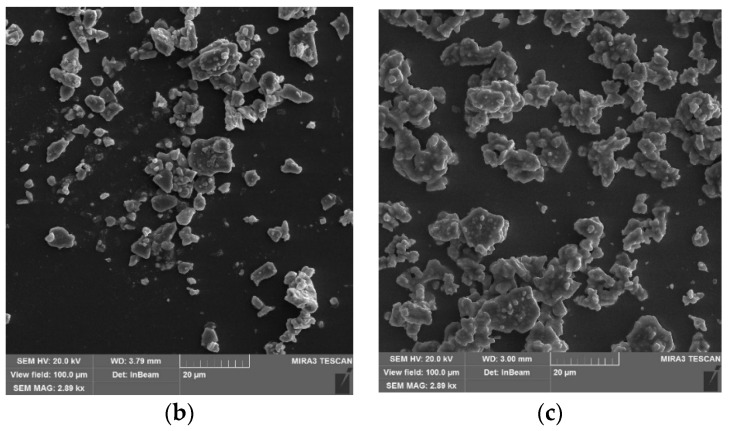
SEM images of multi-walled carbon nanotubes (**a**) and BaFe_12–*x*_Ga*_x_*O_19_ powders: (**b**) x = 0.3; (**c**) x = 0.9.

M-type BaFe_12-x_Ga_x_O_19_ (x = 0.1–1.2) hexagonal ferrites were prepared by the method of solid-state reaction. High-purity Ga_2_O_3_ and Fe_2_O_3_ oxides and BaCO_3_ carbonate were used in a stoichiometric ratio [25]. The synthesis was conducted at 1200 °C for 6 h. Epoxy-based composite materials (CMs) with a magnetic nanofiller (BaFe_12–x_Ga_x_O_19_) and carbon nanotubes (CNTs) were prepared by the method of mixing in solution. Multi-walled carbon nanotubes (CNTs, length of 10–30 μm, outer diameter of 10–30 nm) were purchased from CheapTubes Ins, (Grafton, WV, USA) (Figure 1a). Low-viscosity epoxy resin Larit285 (abbreviated L285) (Lange&Ritter, Gerlingen, Germany) with hardening agent H285 was used as a polymer matrix. The main stages of the investigated CMs’ preparation were as follows. A mixture of L285 epoxy resin and appropriate BaFe_12-x_Ga_x_O_19_ (x = 0.1−1.2) powder was subjected to initial ultrasound action (in a BAKU 9050 ultrasonic cleaner, Guangzhou Hanker Electronics Technology Co., Ltd., Guangzhou, China, 40 kHz, 50 W), for 1 h. In the case of CMs with a nanocarbon component, CNTs were then added, and the mixture was ultrasonicated for an extra hour. After addition of H285, the liquid composite mixture was carefully mixed and then poured into a mold made of a nonmagnetic silicon material. Further, for CMs with a uniform filler distribution, the samples were polymerized under normal conditions in air for one day, followed by drying of the cured CMs at a stepwise increasing temperature from 40 to 800 °C for 5 h. As for the CMs with an aligned BaFe_12−x_Ga_x_O_19_ (x = 0.1–1.2) filler distribution in the polymer matrix, alignment was performed by the placement of a mold containing a liquid CM mixture in a magnetic field of ~0.64 T. Molds were left in the magnetic field until full epoxy polymerization was achieved, followed by drying (according to the above-described scheme).

Figure 1 displays the scanning electron microscopy images of CNTs and BaFe_12–*x*_Ga*_x_*O_19_ fillers.

As can be seen from Figure 1b,c, a certain dispersion of particle sizes was observed for BaFe_12–*x*_Ga*_x_*O_19_ powders with Ga^3+^ concentrations of x = 0.3 and 0.9. The size of BaFe_11.7_Ga_0.3_O_19_ particles changes in the range 0.5–12 μm, the average particle size is 6 μm, and some agglomerates of barium hexaferrite particles are observed. In the case of BaFe_11.1_Ga_0.9_O_19_ powders, the particles’ size is slightly higher (0.7–14 μm, average size is 7 μm), and a larger number of agglomerated barium hexaferrite particles are formed.

Epoxy composites with the combined filler CNT/BaFe_12–*x*_Ga*_x_*O_19_ were prepared. A detailed description of the composite fabrication method with random and aligned filler distributions was presented in our previous paper [26]. The use of ultrasonic dispersion of the composite mixture allows de-agglomeration of the barium hexaferrite filler and a uniform distribution of fillers in the epoxy matrix. The contents of fillers in epoxy composites were as follows: BaFe_12–*x*_Ga*_x_*O_19_–30 wt.%, CNT–1 wt.%.

Microwave scalar network analyzers P2-67 within a 36–55.5 GHz frequency range were used for measurements of the standing wave ratio (SWR) and transmission coefficient T of the investigated CMs at room temperature. Measurements using scalar network analyzers were performed for specimens with dimensions of 5.2 × 2.6 × 2.6 mm^3^.

The measurement configuration was such that the direction of alignment of the filler in the sample was across the direction of the incident wave. The shielding effectiveness $SE_{T}$ (in dB) is related to the measured EMR transmission index *T* using the following equations:

(1) $$SE_{T}=10\log T$$

where $T={\left|E_{T}/E_{I}\right|}^{2}$, $E_{I}$, $E_{T}$ are the electric field strengths of the incident and transmitted waves.

## 3. Results and Discussion

### 3.1. Amplitude-Frequency Characteristics of NFMR

**Figure 2:**
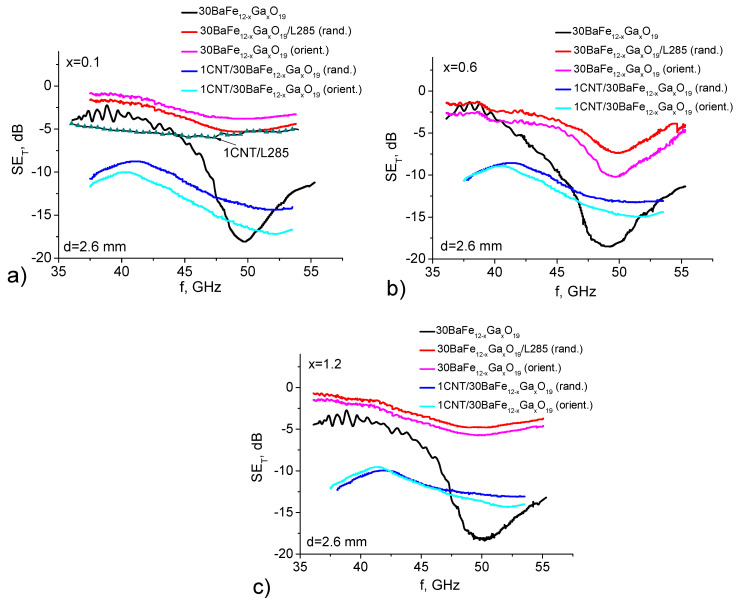
EMR transmission spectra for epoxy composites filled with substituted BaFe_12–*x*_Ga*_x_*O_19_: (**a**) x = 0.1; (**b**) x = 0.6; (**c**) x = 1.2; curve marked by symbols corresponds to 1 wt.% CNT/epoxy CM.

The frequency dependencies of the electromagnetic response (shielding efficiency $SE_{T}$) for epoxy composites with BaFe_12–*x*_Ga*_x_*O_19_ and 1% BHT/BaFe_12–*x*_Ga*_x_*O_19_ for random and oriented distributions of fillers are shown in Figure 2, which also shows the curve of $SE_{T}(f)$ for the 1% CNT/epoxy composite [27,28] and pressed BaFe_12–*x*_Ga*_x_*O_19_ samples for comparison. As can be seen from the figure, at the frequency f≈ 50 GHz, there is a minimum on the $SE_{T}(f)$ curve for all studied samples corresponding to the lower-order natural ferromagnetic resonance (NFMR) modes. The minimum of $SE_{T}$ is most clearly pronounced for pressed powders of nanocrystalline BaFe_12–*x*_Ga*_x_*O_19_, although it is much wider than that observed for single crystal samples [7]. For 30 wt.% BaFe_12–*x*_Ga*_x_*O_19_/epoxy composites, the value of $\left|SE_{T\min}\right|$ is sufficiently lower and less pronounced in comparison with pressed samples of BaFe_12–*x*_Ga*_x_*O_19_, which is explained by the small volume content of BaFe_12–*x*_Ga*_x_*O_19_ (~8.5 vol.%).

The addition of 1 wt.% of CNTs to 30 wt.% BaFe_12–*x*_Ga*_x_*O_19_/epoxy composites leads to a significant increase in EMR shielding $SE_{T}$; however, the shape of the $SE_{T}(f)$ curves with a wide minimum changes only slightly. As it is known, the main parameters that are responsible for the excellent EMR shielding properties of the materials are their electrical conductivity σ and electrodynamic parameters, such as complex permittivity $\varepsilon_{r}^{*}={\varepsilon^{\prime }}_{r}-i{\varepsilon^{\prime \prime }}_{r}$ and magnetic permeability $\mu_{r}^{*}={\mu^{\prime }}_{r}-i{\mu^{\prime \prime }}_{r}$.

The EMR shielding efficiency $SE_{T}$ (in dB) is defined by the following expression [29,30]:

(2) $$\begin{array}{l} SE_{T}=20\mathrm{lg}\left|t\right|=-20\mathrm{lg}\left|e^{\gamma \cdot d}\right|-20\mathrm{lg}\frac{\left|{\left(1+n\right)}^{2}\right|}{4\left|n\right|}-20\mathrm{lg}\left|1-\frac{{\left(1-n\right)}^{2}}{{\left(1+n\right)}^{2}}\cdot e^{-2\gamma \cdot d}\right|\\ =SE_{A}+SE_{R}+SE_{I}\overset{}{,} \end{array}$$

where $SE_{A}$ is the shielding factor due to the EMR absorption; $SE_{R}$ and $SE_{I}$ are the shielding factors due to reflection and multiple reflection, respectively; $n=k_{z}/k_{0}$ is the complex index of refraction; $k_{0}=2\pi /\lambda_{0}$ is the wave vector in free space; $\lambda_{0}=C_{0}/f$; $\lambda_{0}$ and f are the wavelength and the frequency; $C_{0}$ = 3 × 10^8^ m/s; $k_{z}=k_{0}\cdot \sqrt{\varepsilon_{r}^{*}\mu_{r}^{*}}$; $\gamma =i\cdot k_{z}=\alpha +i\beta$ is the propagation constant of the electromagnetic waves; β is the phase constant; α is the attenuation index; and d is the sample thickness.

The higher the electrical conductivity—and, accordingly, the imaginary part of the dielectric permittivity ${\varepsilon^{\prime \prime }}_{r}^{}=\sigma /\left(\omega \cdot \varepsilon_{0}\right)$—the higher the degree of EMR shielding, both due to the high reflection coefficient and effective absorption of EMR. It is obvious that the introduction of highly conductive carbon nanotubes into the polymer matrix leads to an increase in the electrical conductivity of the material and, accordingly, to a weakening of EMR. Table 1 presents data on electrical conductivity for various composites with fillers of CNTs, BaFe_12–*x*_Ga*_x_*O_19_, and CNT/BaFe_12–*x*_Ga*_x_*O_19_.

As can be seen from the presented data, the addition of 1% CNTs to epoxy leads to an increase in electrical conductivity, but the percolation threshold has not yet been reached. The introduction of BaFe_12–*x*_Ga*_x_*O_19_ alone does not lead to significant changes in electrical conductivity, since BaFe_12–*x*_Ga*_x_*O_19_ is a dielectric. Moreover, as can be seen from Figure 2a and Table 1, the $SE_{T}$ values correlate with the data on the electrical conductivity of these CMs: $SE_{T}$ is minimal for epoxy and BaFe_12–*x*_Ga*_x_*O_19_ and increases for 1% CNT/epoxy, since the electrical conductivity and complex dielectric permittivity $\varepsilon_{r}^{*}$ increase, especially the imaginary part of the dielectric permittivity ${\varepsilon^{\prime \prime }}_{r}^{}=\sigma /\left(\omega \cdot \varepsilon_{0}\right)$, which is responsible for the absorption of EMR.

The use of a combined filler, 1% CNT/30% BaFe_12–*x*_Ga*_x_*O_19_, leads to a further slight increase in electrical conductivity (up to 5.0 × 10^−8^ S/m) compared to 1% CNT/epoxy CM; however, a significant increase in shielding $SE_{T}$ is observed. Such an increase in $SE_{T}$ for ternary CMs is related not only to increased conduction loss but also to the occurrence of magnetic loss due to the presence of magnetic particles of BaFe_12–*x*_Ga*_x_*O_19_. In addition, it may be assumed that the use of CNT and BaFe_12–*x*_Ga*_x_*O_19_ fillers in combination results in an increase in the real part of dielectric permittivity ${\varepsilon^{\prime }}_{r}$ due to the formation of a large number of dipoles and strong interfacial polarization [32,33]. This increase in ${\varepsilon^{\prime }}_{r}$ promotes an increase in shielding due to the reflection of EMR. Thus, the use of CNTs in combination with magnetic BaFe_12–*x*_Ga*_x_*O_19_ particles as fillers in epoxy matrices results in an increase in dielectric permittivity ${\varepsilon^{\prime }}_{r}$, a slight increase in magnetic permeability ${\mu^{\prime }}_{r}$, and also an increase in dielectric ${\varepsilon^{\prime \prime }}_{r}$ and magnetic ${\mu^{\prime \prime }}_{r}$ losses. Such changes in electrodynamic parameters of CMs lead to an increase in the EMR attenuation coefficient α, which is responsible for the attenuation of incident electromagnetic radiation [32]:

(3) $$\alpha =\frac{\sqrt{2}\pi f}{C}\sqrt{\left(\mu^{\prime \prime }\varepsilon^{\prime \prime }-\mu^{\prime }\varepsilon^{\prime }\right)+\sqrt{{\left(\mu^{\prime \prime }\varepsilon^{\prime \prime }-\mu^{\prime }\varepsilon^{\prime }\right)}^{2}+{\left(\mu^{\prime }\varepsilon^{\prime \prime }+\mu^{\prime \prime }\varepsilon^{\prime }\right)}^{2}}}$$

where C is the velocity of light.

The high dielectric ${\varepsilon^{\prime \prime }}_{r}$ and magnetic loss ${\mu^{\prime \prime }}_{r}$ could result in a high value of α.

**Figure 3:**
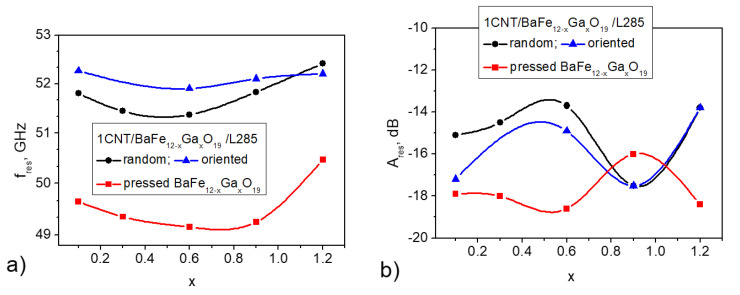
Concentration dependences of resonance NFMR frequency $f_{res}$ (**a**) and resonance NFMR amplitude $A_{res}$ (**b**) for epoxy composites with 1% CNT/BaFe_12-*x*_Ga*_x_*O_19_ (random and oriented filler distributions) and initial pressed sample BaFe_12−*x*_Ga*_x_*O_19_.

Figure 3 displays the resonance frequency of NFMR for various types of composites with BaFe_12–*x*_Ga*_x_*O_19_. The NFMR frequency $f_{res}$ was measured at half of the bandwidth $W_{res}/2$. As can be seen in Figure 3, the frequency of the NFMR resonance for epoxy composites containing 1 wt.%CNT/30 wt.% BaFe_12–*x*_Ga*_x_*O_19_ is higher compared with the pressed BaFe_12–*x*_Ga*_x_*O_19_ sample, and such a change is similar to the 30 wt.%BaFe_12–*x*_Ga*_x_*O_1_/epoxy composites investigated in our previous paper [34].

$f_{res}$ is determined by the magneto-crystalline anisotropy field $H_{a}$ and magnetic saturation $M_{s}$ of BaFe_12–*x*_Ga*_x_*O_19_ [35,36]:

(4) $$f_{res}=\frac{\gamma }{2\pi }\left(H_{a}-4\pi M_{s}\right)$$

where γ/2π = 2.8 MHz/Oe is the gyromagnetic ratio.

Following from Equation (4), the increase in $f_{res}$ may be related to the increase in $H_{a}$ at $M_{s}$ = constant or to the decrease in $M_{s}$ at $H_{a}$ = const. It may be concluded that the increase in $f_{res}$ in the case of the 1% CNT/30 wt.% BaFe_12–*x*_Ga*_x_*O_19_/epoxy composite is the result of the $H_{a}$ increase and $M_{s}$ decrease observed for 30 wt.% BaFe_12–*x*_Ga*_x_*O_19_/epoxy CMs in our previous research [37]; it was shown that the magnetic parameters of 30 wt.% BaFe_12–*x*_Ga*_x_*O_19_/epoxy composites are higher than the corresponding parameters of pure BaFe_12–*x*_Ga*_x_*O_19_ (0 ≤ x ≤ 0.1) polycrystalline samples. It was concluded that the polymer coating on magnetic particles obviously affects the contributions of the surface anisotropy, shape anisotropy, and interface anisotropy to the total anisotropy [38,39]. The slightly higher values of $f_{res}$ for the aligned 1% CNT/30 wt.% BaFe_12–*x*_Ga*_x_*O_19_/epoxy composite may be related to the higher value of the magneto-crystalline anisotropy field $H_{a}$ due to a change in the shape anisotropy at the formation of the elongated barium hexaferrite chains under magnetic field alignment [36,40].

This $f_{res}$ also depends on the cation Ga^3+^ concentration in BaFe_12–*x*_Ga*_x_*O_19_. The concentration dependencies of the resonance frequency for BaFe_12–*x*_Ga*_x_*O_19_ and 1% CNT/30% BaFe_12–*x*_Ga*_x_*O_19_/epoxy are nonmonotonic and have a minimum at *x* = 0.6. As shown for BaFe_12–*x*_Ga*_x_*O_19_, this dependence can be satisfactorily approximated by the second-order polynomial $f_{res}=50.04+3.37x^{2}-3.73x$ [41]. This concentration behavior is observed during a monotonic decrease in the magnetic parameters, such as the Curie temperature, the remnent magnetization, and the coercive force, when the cation Ga^3+^ concentration increases. Thus, the increase in the resonance frequency at x ≥ 0.6 is thought to be caused by an increase in the magneto-crystalline anisotropy field $H_{a}$ and a decrease in the saturation magnetization $M_{s}$ with the Ga^3+^ content increase. For 1% CNT/30% BaFe_12–*x*_Ga*_x_*O_19_/epoxy CMs with an oriented distribution of fillers, the dependence of $f_{res}$ on the Ga^3+^ concentration is weaker than for random 1% CNT/30% BaFe_12–*x*_Ga*_x_*O_19_/epoxy CMs.

The amplitude of the resonance for 1% CNT/30% BaFe_12–*x*_Ga*_x_*O_19_/L285 is lower compared with a pure pressed sample of BaFe_12–*x*_Ga*_x_*O_19_ and also changes with the Ga^3+^ concentration: firstly, it decreases with the Ga^3+^ concentration up to x = 0.6, and then it sharply increases for x = 0.9 and decreases again for x = 1.2. For the pressed samples of BaFe_12–*x*_Ga*_x_*O_19_, the opposite behavior of $A_{res}$ on the Ga^3+^ concentration is observed. It should be noted that the determination of $A_{res}$ for 1% CNT/30% BaFe_12–*x*_Ga*_x_*O_19_/L285 is approximate, since the resonance peaks are less pronounced.

### 3.2. Amplitude-Frequency Characteristics of NFMR for 1% CNT/30% BaFe_12-x_Ga_x_O_19_/Epoxy Composites at Applied Magnetic Field

**Figure 4:**
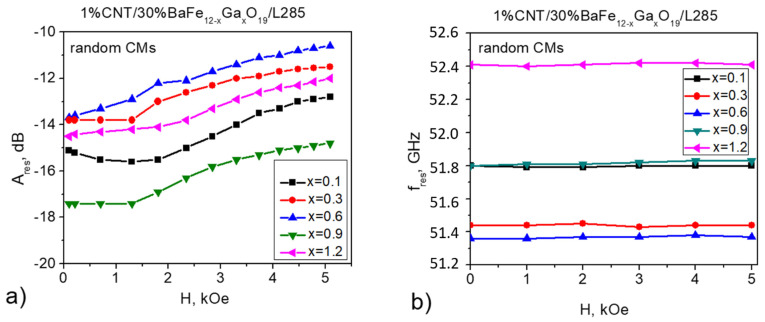
FMR amplitude (**a**) and frequency (**b**) as a function of the applied external magnetic field measured for random 1% CNT/30% BaFe_12–*x*_Ga*_x_*O_19_/L285 composites.

**Figure 5:**
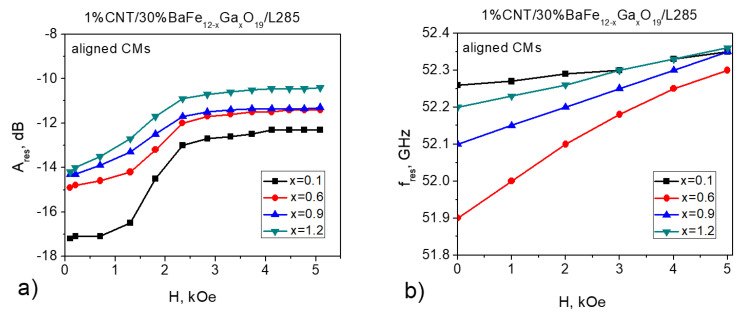
FMR amplitude (**a**) and frequency (**b**) as a function of the applied external magnetic field measured for aligned 1% CNT/30% BaFe_12–*x*_Ga*_x_*O_19_/L285 composites.

Figure 4 and Figure 5 present the results of the NFMR study in which a DC magnetic field was applied to 1% CNT/30% BaFe_12–*x*_Ga*_x_*O_19_/L285. As can be seen from Figure 4a, an applied DC magnetic field leads to a decrease in the amplitude of the NFMR resonance for the 1% CNT/30% BaFe_12–*x*_Ga*_x_*O_19_/L285 composite with a random filler distribution for all Ga^3+^ concentrations (x = 0.1–1.2). Regarding the frequency of the NFMR resonance, this does not change with the application of a DC magnetic field.

In the case of 1% CNT/30% BaFe_12–*x*_Ga*_x_*O_19_/L285 with an aligned filler distribution (Figure 5a), an increase in the amplitude of NFMR was also observed; however, the $A_{res}(H_{ext})$ dependencies are more complicated. Firstly, $A_{res}$ increases with $H_{ext}$ up to 2.5 kOe and then does not change with the magnetic field increase. Contrary to the random 1% CNT/30% BaFe_12–*x*_Ga*_x_*O_19_/L285 composite, for the aligned 1% CNT/30% BaFe_12–*x*_Ga*_x_*O_19_/L285 composites, a slight increase in $f_{res}$ is observed. For example, for 1% CNT/30% BaFe_12–*x*_Ga*_x_*O_19_/L285 with 0.6 Ga^3+^, $f_{res}$ increases from 51.9 to 52.3 GHz in the $H_{ext}$ range 0–5 kOe.

It was noted that such an increase in $f_{res}$ was sufficiently lower compared with pressed polycrystalline BaFe_12–*x*_Ga*_x_*O_19_, where $f_{res}$ increased from 49 to 54 GHz in the $H_{ext}$ range 0–3.5 kOe; these dependencies were almost linear for all samples [40]. As concluded in [40] for pressed polycrystalline BaFe_12–*x*_Ga*_x_*O_19_, the resonance frequency increased with the magnetic field as the internal magnetic field related to the anisotropy increased.

Such behavior of the minimums of the $SE_{T}(f)$ dependencies and changes in the amplitudes of the $SE_{T}$ peaks with the variation in the magnetic field values confirms their ferromagnetic nature.

Within the theory of hexagonal ferrites, the NFMR frequency $f_{res}$ at the applied external magnetic field $H_{ext}$ may be described by the following expression [42]:

(5) $$f_{res}=\frac{\gamma }{2\pi }\left(H_{ext}+H_{a}-4\pi M_{s}\right)$$

where $H_{ext}$ is the applied DC magnetic field.

As shown in [41] for BaAl*_x_*Fe_12–*x*_O_19_ samples, the behavior of the resonance frequency $f_{res}$ versus the applied magnetic field $H_{ext}$ is determined by the value of the saturation magnetic field $H_{sat}$. For the range of the external magnetic field $H_{ext}<H_{sat}$, the resonance frequency $f_{res}$ is approximately constant at the applied DC magnetic field. If the value of the applied magnetic field $H_{ext}$ is higher than $H_{sat}$, $f_{res}$ of NFMR linearly increases with $H_{ext}$. Table 2 shows the data on magnetic parameters for the pressed polycrystalline BaFe_12–*x*_Ga*_x_*O_19_ samples and 30 wt.% BaFe_12–*x*_Ga*_x_*O_19_/epoxy composites (x = 0.1–1.2), which were studied in our previous papers [37,40].

As can be seen from Table 2, for the pressed polycrystalline BaFe_12–*x*_Ga*_x_*O_19_ samples, $H_{sat}$≈ 20 kOe [40], which is why the approximately linear dependencies $f_{res}(H_{ext})$ in the range of $H_{ext}$ = (0–4) kOe have a slope (γ/2π = 1.5–2) that is lower than the theoretical value of 2.8.

In the case of 30% BaFe_12–*x*_Ga*_x_*O_19_/L285 (for x = 0–1.2), the values of the saturation magnetic field $H_{sat}$ are higher (23–33) kOe [39], which results in independence (for random composites) or only a slight increase (for aligned epoxy CMs with a lower saturation field $H_{sat}$ compared with random composites) in $f_{res}$ with the applied magnetic field $H_{ext}$. This statement is also correct for both random and aligned 1% CNT/30% BaFe_12–*x*_Ga*_x_*O_19_/L285 composites. Thus, we need to highlight that the quality of the dispersion/alignment of fillers in composites is very important for the electrodynamic properties of CMs [42,43].

## 4. Conclusions

Epoxy composites with random and aligned magnetic field distributions of 1 wt.% of CNTs and 30 wt.% of BaFe_12–*x*_Ga*_x_*O_19_ (0.1 < *x* < 1.2) were fabricated. It was found that adding 1 wt.% CNTs along with 30 wt.% of BaFe_12–*x*_Ga*_x_*O_19_ into epoxy resin resulted in an increase in electrical conductivity up to 5.0 ×⋅10^−8^ S/m that is explained by the high electrical conductivity of CNTs. The observed sufficient increase in the microwave shielding efficiency of ternary random and aligned 1% CNT/30 wt.% BaFe_12–*x*_Ga*_x_*O_19_/epoxy composites in the frequency range 36–55 GHz compared with binary 1% CNT/epoxy and 30 wt.% BaFe_12–*x*_Ga*_x_*O_19_/epoxy was explained by the increased complementary contributions of dielectric and magnetic losses and the increase in the EMR attenuation constant. The higher values of the natural ferromagnetic resonance (NFMR) frequency $f_{res}$ (51.8–52.4 GHz) and weaker dependence of $f_{res}$ on the Ga^3+^ concentration in 1 wt.% CNT/30 wt.% BaFe_12–*x*_Ga*_x_*O_19_/epoxy composites compared with pressed polycrystalline BaFe_12–*x*_Ga*_x_*O_19_ ($f_{res}$ = 49.6–50.4 GHz) may be related to the higher values of the magnetic parameters of 30 wt.%BaFe_12–*x*_Ga*_x_*O_19_ in the epoxy matrix compared with the corresponding parameters of pure pressed BaFe_12–*x*_Ga*_x_*O_19_ (0 ≤ x ≤ 0.1) polycrystalline samples. The slightly higher values of $f_{res}$ for the aligned 1% CNT/30 wt.% BaFe_12–*x*_Ga*_x_*O_19_/epoxy composite compared with the random composites may be related to the higher value of the magneto-crystalline anisotropy field $H_{a}$ due to a change in the shape anisotropy at the formation of the elongated barium hexaferrite chains under magnetic field alignment. The approximately constant value of the NFMR frequency $f_{res}$ in the range of the applied magnetic field, H = 0–5 kOe, for the random 1 wt.% CNT/30 wt.% BaFe_12–*x*_Ga*_x_*O_19_/epoxy composite and slightly increased $f_{res}$ for the aligned 1 wt.% CNT/30 wt.% BaFe_12–*x*_Ga*_x_*O_19_/epoxy composite were explained by the lower saturation field $H_{sat}$ compared to the pressed polycrystalline BaFe_12–*x*_Ga*_x_*O_19_ samples. The obtained results open up the prospect for practical applications of such materials in antenna technologies (as well as 5G).

## Figures and Tables

**Table 1:** Electrical conductivity, shielding efficiency, dielectric permittivity, and magnetic permeability for epoxy composites with various fillers.

Composite Material	σ, S/m	$SE_{T},\;\mathrm{dB}$	${\varepsilon^{\prime }}_{r}$	${\varepsilon^{\prime \prime }}_{r}$	${\mu^{\prime }}_{r}$	Ref.
		*f* = 40 GHz, d = 2.6 mm				
Epoxy resin L285	1.0 × 10^−11^	−1	2.9	0.008	1	[28]
30%BaFe_12_O_19_/L285	1.0 × 10^−10^	−1	4.0	0.21	1.45	[31]
1%CNT/L285	2.0 × 10^−8^	−4	3.8	0.57	1	[27,28]
1%CNT/30%BaM/L285	5.0 × 10^−8^	−10	-	-		This work

**Table 2:** The magnetic parameters of pressed BaFe_12–*x*_Ga*_x_*O_19_ samples and 30 wt.% BaFe_12–*x*_Ga*_x_*O_19_/epoxy composites (x = 0.1–1.2) with random and aligned distributions of fillers in the epoxy matrix.

Composite	Filler Distribution	*H_c_*, kA/m	$M_{s},\;A\;m^{2}\;{\mathrm{kg}}^{-1}$	$H_{sat},\;\mathrm{kA}/m$	Ref.
Pressed polycrystalline BaFe_11.9_Ga_0.1_O_19_	-	175.07	56	~1591.549	[40]
Pressed polycrystalline BaFe_11.4_Ga_0.6_O_19_	-	59.683	46	~1591.549	[40]
Pressed polycrystalline BaFe_10.8_Ga_1.2_O_19_	-	39.788	30	~1591.549	[40]
30 wt.% BaFe_11.9_Ga_0.1_O_19_/epoxy	random aligned	47.746 45.359	18.84 28.37	2641.972 1901.901	[39]
30 wt.% BaFe_11.4_Ga_0.6_O_19_/epoxy	random aligned	85.943 81.964	17.53 20.32	2570.357 2347.535	[39]
30 wt.% BaFe_10.8_Ga_1.2_O_19_/epoxy	random aligned	132.098 126.528	14.94 14.72	2514.648 2299.788	[39]

## Data Availability

Data is contained within the article.
